# *E. coli* HMS174(DE3) is a sustainable alternative to BL21(DE3)

**DOI:** 10.1186/s12934-018-1016-6

**Published:** 2018-10-30

**Authors:** Johanna Hausjell, Julia Weissensteiner, Christian Molitor, Heidi Halbwirth, Oliver Spadiut

**Affiliations:** Research Division Biochemical Engineering, Institute of Chemical, Environmental and Bioscience Engineering, TU Wien, Vienna, Austria

**Keywords:** *E. coli*, Recombinant protein production, IPTG, Lactose, BL21(DE3), HMS174(DE3)

## Abstract

**Background:**

*Escherichia coli* is one of the most widely used hosts for recombinant protein production in academia and industry. Strain BL21(DE3) is frequently employed due to its advantageous feature of lacking proteases which avoids degradation of target protein. Usually it is used in combination with the T7-pET system where induction is performed by one point addition of IPTG. We recently published a few studies regarding lactose induction in BL21(DE3) strains. BL21(DE3) can only take up the glucose-part of the disaccharide when fed with lactose. However, initially additional glucose has to be supplied as otherwise the ATP-related lactose uptake barely happens. Yet, as lactose is an inexpensive compound compared to glucose and IPTG, a new induction strategy by a lactose-only feed during induction seems attractive. Thus, we investigated this idea in the galactose metabolizing strain HMS174(DE3).

**Results:**

We show that strain HMS174(DE3) can be cultivated on lactose as sole carbon source during induction. We demonstrate that strain HMS174(DE3) exhibits higher product and biomass yields compared to BL21(DE3) when cultivated in a lactose fed-batch. More importantly, HMS174(DE3) cultivated on lactose even expresses more product than BL21(DE3) in a standard IPTG induced glucose fed-batch at the same growth rate. Finally, we demonstrate that productivity in HMS174(DE3) lactose-fed batch cultivations can easily be influenced by the specific lactose uptake rate (q_s,lac_). This is shown for two model proteins, one expressed in soluble form and one as inclusion body.

**Conclusions:**

As strain HMS174(DE3) expresses even slightly higher amounts of target protein in a lactose fed-batch than BL21(DE3) in a standard cultivation, it seems a striking alternative for recombinant protein production. Especially for large scale production of industrial enzymes cheap substrates are essential. Besides cost factors, the strategy allows straight forward adjustment of specific product titers by variation of the lactose feed rate.

**Electronic supplementary material:**

The online version of this article (10.1186/s12934-018-1016-6) contains supplementary material, which is available to authorized users.

## Background

*Escherichia coli* is one of the most widely used hosts for the production of recombinant protein [1–5]. It thus plays a prominent role in research as well as in industry where it serves as host for the production of more than 30% of approved therapeutic proteins [3, 6]. Its advantages result from comprehensive knowledge about the prokaryote coming along with many and comparably fast tools for genetic manipulation [1, 5]. Furthermore, it can be cultivated on inexpensive media up to high cell densities allowing exceptionally high product titers [2, 7].

The most frequently used strain for heterologous protein production in *E. coli* is strain BL21(DE3) as it offers several convenient features including the fact that it lacks the Lon and the OmpT proteases [1, 8]. Most frequently, strain BL21(DE3) is employed together with the T7 expression system [9]. The system is based on the T7 promoter, which features exceptionally high transcription rates as the target protein is transcribed by the T7 polymerase which is faster compared to native *E. coli* polymerases [1, 10, 11]. Conventionally, the T7 system is induced by one point addition of IPTG [10, 12]. However, IPTG has several drawbacks as it puts a high metabolic burden on the organism and is associated with inclusion body formation [13–16]. Thus, our research group recently published several studies using the comparably inexpensive disaccharide lactose as alternative inducer for BL21(DE3) strains. We showed that productivity, product location and inclusion body properties can be stirred via the specific glucose and lactose uptake rates during induction [17–20].

However, strain BL21(DE3) carries deletions of galactokinase, galactose-1-phosphate uridylyltransferase and UDP-glucose 4-epimerase, enzymes which are important in the galactose utilization or Leloir pathway. Therefore, this strain is not able to metabolize galactose. In one of its ancestors in the B-Line, B707, this mutation was introduced by P1 transduction of WA628 from Bc258, which is a non-reverting Gal^−^ mutant that was obtained by UV radiation [21]. Thus, galactose accumulates, whenever feeding BL21(DE3) with lactose. Also, additional glucose has to be supplied in limiting amounts as the ATP stemming from the glucose part of the lactose is not sufficient for efficient ATP-related lactose transport into the cells. Simultaneous feeding of glucose and lactose leads to a rather complex correlation of their uptake rates which was also shown to be product dependent [17, 18].

In contrast to BL21(DE3), strains JM109(DE3) and HMS174(DE3) are both able to metabolize galactose. They stem from the *E. coli* K-12 line and do not harbor mutations in their galactose pathway [22, 23]. Both also carry the λ prophage in their genome, allowing recombinant protein production from pET vectors [24].

Recombinantly produced enzymes are needed in many fields, ranging from medicine to food and nutrition or the production of detergents, textiles, leather, paper, pulp and plastics [25]. Especially for industrial applications there is an urge for cheap protein production, as the enzymes sell at comparably low prices. This makes the idea of using lactose as sole carbon source during induction especially interesting: The inexpensive waste product could potentially serve as both, inducer and C-source replacing the comparatively costly combination of IPTG and glucose [26–28]. Thus, we investigated productivity in lactose fed-batches of HMS174(DE3) and BL21(DE3) and compared it to conventional glucose fed-batches with IPTG induction. Motivated by the results, we looked closer into the correlation between productivity and varying specific lactose uptake rates for two model proteins, one expressed as soluble protein and one as inclusion body.

## Results and discussion

### Lactose uptake in HMS174(DE3), JM109(DE3) and BL21(DE3)

In order to investigate our hypothesis that cultivation on lactose as C-source and inducer was possible when employing the T7 system and strains that are able to metabolize galactose, we initially tested two strains that carried the λ prophage but had no deletions of enzymes in the Leloir pathway: JM109(DE3) and HMS174(DE3). Both strains have previously been evaluated and compared to BL21(DE3) in glucose fed-batch cultivations [22, 29, 30]. However, we wanted to investigate their growth on lactose as sole C-source in bioreactor cultivations. All strains investigated expressed a model protein, namely the plant enzyme flavanone 3-hydroxylase (FHT) of *Malus domestica*, which is a key enzyme in the biosynthesis of common flavonoids [31].

**Fig. 1:**
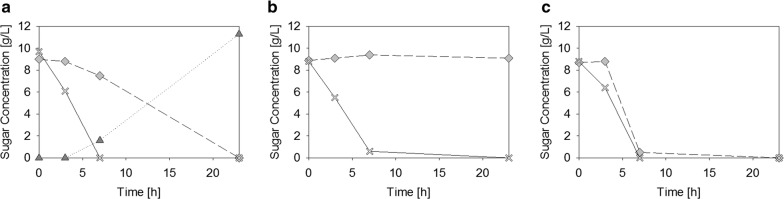
Sugar concentrations in shake-flasks cultivations of **a** BL21(DE3), **b** JM109(DE3) and **c** HMS174(DE3). Sugar-concentrations over time in the supernatant of shake-flask cultivations on DeLisa minimal media with 9 g/L glucose (white crosses) and 9 g/L lactose (grey diamonds). Three cultivations of **a** BL21(DE3), **b** JM109(DE3) and **c** HMS174(DE3) are compared. As BL21(DE3) was the only Gal^−^ strain, accumulation of galactose (dark grey triangles) was observed when lactose was being consumed. The error on sugar measurements is on average 5%

We performed shake flask cultivations with glucose and lactose as carbon sources for BL21(DE3), JM109(DE3) and HMS174(DE3) (Fig. 1).

We observed that BL21(DE3) first took up glucose and then slowly started taking up lactose. This is due to the well-known phenomenon of carbon catabolite repression. Once glucose is taken up, enzyme II A (EIIA) is phosphorylated which activates adenylate cyclase. Consequently, formation of a complex between cyclic adenosine monophosphate receptor protein (CRP) and cyclic adenosine monophosphate (cAMP) is promoted which binds to the promoter region and presents the DNA. This allows facilitated binding of RNA polymerase and thus transcription from the *lac* operon and uptake of the disaccharide (e.g. [32–35]). Figure 1a also shows that galactose accumulated in the media once lactose was taken up. As shown in Fig. 1b JM109(DE3) did not take up lactose even after glucose depletion. We hypothesize that this is because the strain carries a mutated version of the *lac* repressor (LacI^q^) in its genome. This mutation causes higher transcription rates of the *lac* repressor and consequently more repressor-protein (LacI) in the cells [36, 37]. Thus, de-repression, which happens when either lactose or IPTG bind to LacI, cannot happen as easily. HMS174(DE3) on the other hand consumed lactose much faster than BL21(DE3) (Fig. 1c). Already after 7 h, almost all of the disaccharide was gone. We hypothesize that this is due to the fact that HMS174(DE3) is also able to metabolize the galactose-part of the lactose and thus produces more ATP as galactose is introduced into the glycolysis via the Leloir pathway. Thus, lactose uptake can happen faster as more ATP is available. In accordance to these results, production of the model protein was only observed in strains BL21(DE3) and HMS174(DE3) (data not shown).

### Characterization of HMS174(DE3) during lactose induction

**Fig. 2:**
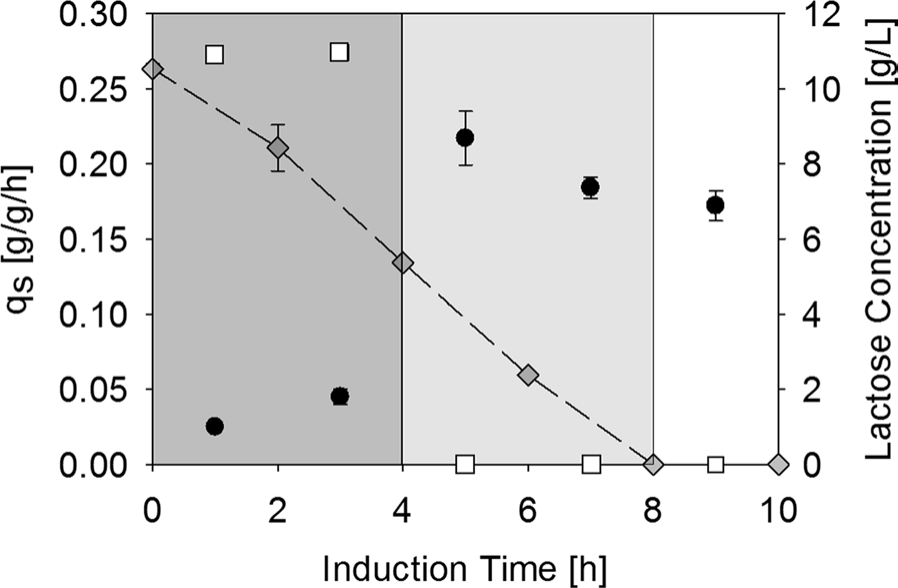
Initial cultivation of HMS174(DE3) on lactose. During the first 4 h (dark grey area) the glucose feed was still running [specific glucose uptake rate = 0.27 g/g/h (white squares)] and lactose was pulsed to 10 g/L (grey diamonds) allowing the cells to adapt to lactose. After 4 h, the glucose feed was switched off, however lactose was still present in excess (light grey area), showing a maximum specific lactose uptake rate (black circles) of 0.23 g/g/h in the absence of glucose. After 8 h of induction, lactose was no longer present in excess

As strain HMS174(DE3) consumed lactose the fastest, it seemed most promising for the lactose-only induction strategy and we decided to investigate it in bioreactor cultivations. From previous experiments with BL21(DE3) we knew that *E. coli* needs approximately 4 h of adaption before being able to take up lactose at its maximum uptake rate [18]. Thus, we carried out a fed batch on glucose for biomass generation and then pulsed lactose to a concentration of 10 g/L while still feeding with a specific glucose uptake rate of 0.27 g/g/h, giving the strain the necessary energy for expressing the enzymes needed for lactose metabolism [18]. After those 4 h, the glucose feed was switched off and a lactose feed was started at a set point of 0.2 g/g/h. The uptake rate of 0.2 g/g/h was an approximate estimation calculated from OD-values and sugar concentrations in the shake-flask experiments. Sugar contents in the supernatant and specific sugar uptake rates during the cultivation are shown in Fig. 2.

As shown in Fig. 2 the maximum specific lactose uptake rate in the presence of glucose within the first 4 h (dark grey area) increased over time as the strain adapted to the disaccharide and went up to 0.045 g/g/h. With lactose in excess and the absence of glucose (light grey area) the maximum specific lactose uptake rate was 0.23 g/g/h. The approximate fivefold increase in the maximum lactose uptake rate in the absence of glucose was a result of the lifted carbon catabolite repression when glucose was no longer supplied. After the first 8 h of induction, the excess lactose was consumed and lactose was fed in limiting amounts of 0.2 g/g/h. Viability measurements after 10 h of induction showed that more than 94% of the cells were alive, an exceptionally high proportion, and production of target protein was observed as well (Additional file 1: Figure S1).

### Comparison of HMS174(DE3) to BL21(DE3) in lactose fed-batches

**Fig. 3:**
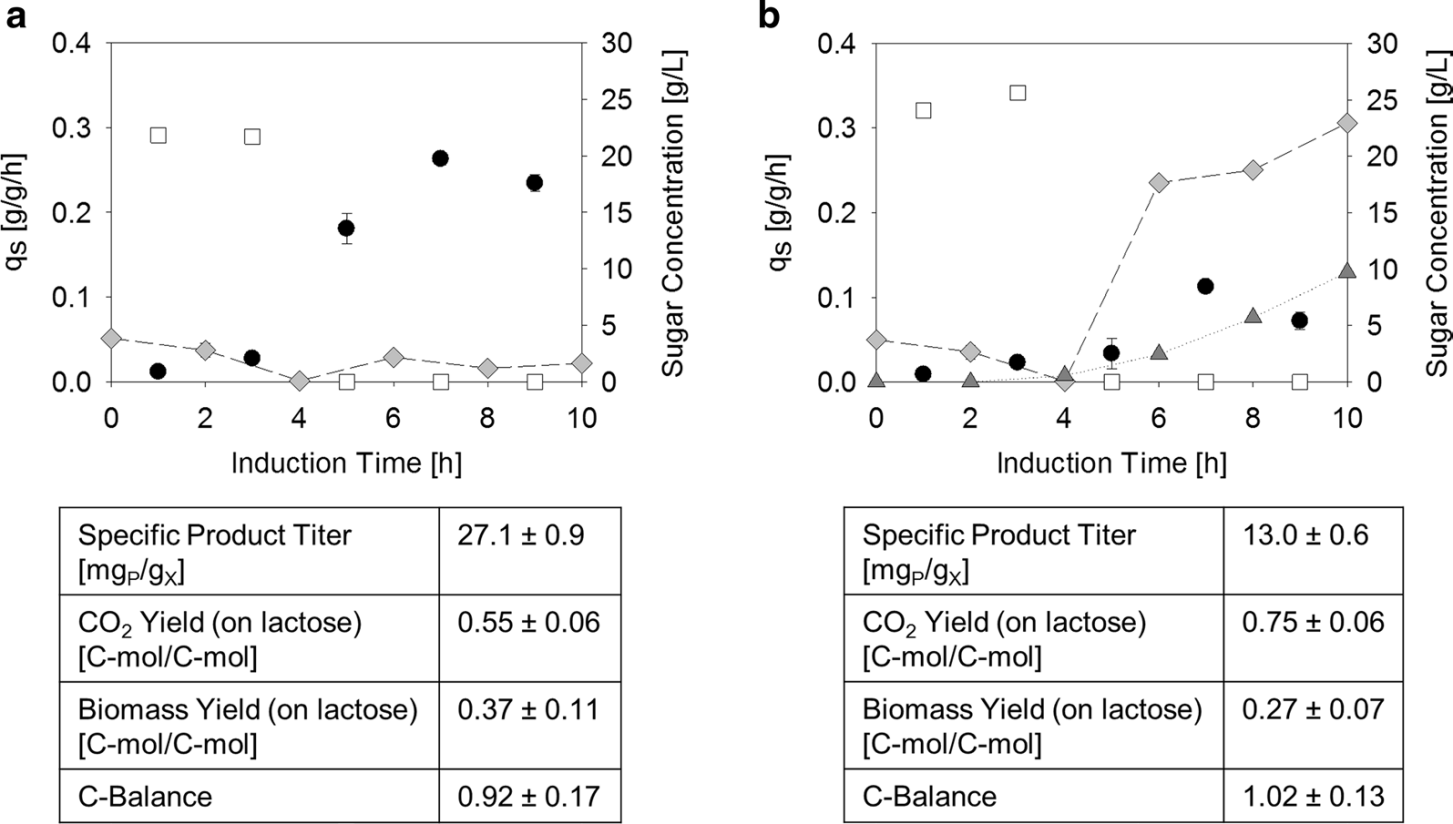
Comparison of **a** HMS174(DE3) and **b** BL21(DE3) on an induced lactose fed-batch regarding productivity of FHT and physiological parameters. Lactose (grey diamonds) was pulsed to 5 g/L while glucose was still fed for 4 h at a specific glucose uptake rate (white squares) of 0.27 g/g/h. After 4 h, the glucose feed was switched off and lactose was depleted. Then a lactose feed was started at a rate that exceeded the maximum specific lactose uptake rate (black dots) so that lactose was always present in excess. In the BL21(DE3) cultivation galactose (dark grey triangles) started to accumulate after the lactose feed was started. FHT production and physiological parameters are shown below and are calculated as average during the 6 h of lactose feeding. Standard deviations were evaluated from triple measurements and calculated by error propagation

We wanted to investigate if strain HMS174(DE3) was in fact superior to strain BL21(DE3) when feeding with lactose as sole C-source during induction. Both strains were cultivated under equal process parameters. Lactose was pulsed to a concentration of 5 g/L 4 h before ending the glucose fed batch allowing the cells to adapt. After the adaption period, a lactose feed at 0.25 g/g/h was started for both strains. The specific sugar uptake rates and the sugar contents in the supernatant as well as physiological data and productivity of both strains are plotted in Fig. 3. Strong accumulation of lactose was observed in the BL21(DE3) cultivation only 2 h after the feed was started. Therefore, the feed-rate was turned down to a setpoint of 0.1 g/g/h in this cultivation. As shown in Fig. 3, BL21(DE3) had an almost 3 times lower maximum lactose uptake rate compared to HMS174(DE3), namely 0.08 g/g/h and 0.23 g/g/h, respectively. We hypothesize that HMS174(DE3) can take up more lactose as utilization of galactose and glucose provides more energy, allowing enhanced ATP related lactose-transport into the cell [34, 38, 39].

In the BL21(DE3) cultivation (Fig. 3b) galactose accumulation was observed during lactose feeding as the sugar was not metabolized. This is disadvantageous as high sugar concentrations in the supernatant can expose the cells to osmotic stress [40]. Another striking difference between HMS174(DE3) and BL21(DE3) was found in the physiological data, as HMS174(DE3) converted more lactose to biomass and less to CO_2_. Lastly, but most importantly, also the specific product titer was twice as high in strain HMS174(DE3) as in BL21(DE3). All of this clearly demonstrates that the galactose-metabolizing strain HMS174(DE3) is superior to BL21(DE3) when cultivated on lactose as sole carbon source.

### Comparison of a lactose fed batch of HSM174(DE3) to an IPTG-induced glucose fed batch of BL21(DE3)

The question remained, if the lactose induction strategy in HMS174(DE3) could also compete with IPTG induction in BL21(DE3) on glucose. For comparability reasons the specific sugar uptake rates of glucose and lactose were adjusted in a way that the same specific growth rate was reached in both cultivations. Strain BL21(DE3) was induced with 0.5 mM IPTG.

As shown in Table 1, we found that at the same growth rate of 0.07 h^−1^, similar specific product titers were observed for both strains, the one in HMS174(DE3) even being slightly higher (27 mg product/g DCW in HMS174(DE3) versus [25 mg product/g DCW in BL21(DE3)]. This clearly argues for the lactose induction strategy in HMS174(DE3) as it is a rather inexpensive and sustainable alternative to glucose and IPTG [26–28].

**Table 1:** Comparison of the productivity of FHT in BL21(DE3) and HMS174(DE3)

	q_s_ (g/g/h)	µ (h^−1^)	Specific titer (mg_P_/g_X_)
HMS174(DE3)	q_s,lac,max_ = 0.23 ± 0.034	0.07 ± 0.015	27 ± 0.9
BL21(DE3)	q_s,glu_ = 0.28 ± 0.046	0.07 ± 0.015	25 ± 0.6

A standard BL21(DE3) cultivation on glucose, induced at 0.5 mM IPTG is compared to a HMS174(DE3) cultivation induced and fed with lactose. Standard deviations were evaluated from triple measurements and calculated by error propagation

### Productivity in dependence on induction conditions

Due to these promising results we investigated the correlation between productivity and specific lactose uptake rate in more detail. Stirring productivity by simply adjusting the feed-rate during induction would offer a straight forward tool for regulation of product titers. We studied the effect of q_s,lac_ on the production of two model proteins: FHT of *M. domestica*, which we found to only express as soluble protein in *E. coli*, and chalcone-3-hydroxylase (CH3H) of *Dahlia variabilis*, which primarily formed inclusion bodies. The results are shown in Table 2.

**Table 2:** FHT and CH3H production in dependence on specific lactose uptake rates

FHT—expressed as soluble protein			CH3H—expressed as inclusion body		
µ (h^−1^)	q_s,lac_ (g/g/h)	Specific product titer (mg_P_/g_X_)	µ (h^−1^)	q_s,lac_ (g/g/h)	Specific product titer (mg_P_/g_X_)
0.07 ± 0.015	0.23 ± 0.034	27 ± 0.9	0.08 ± 0.021	0.23 ± 0.038	390 ± 31
0.04 ± 0.014	0.12 ± 0.004	17 ± 1.2	0.04 ± 0.012	0.13 ± 0.005	240 ± 16
0.02 ± 0.001	0.055 ± 0.004	11 ± 0.3	0.02 ± 0.009	0.074 ± 0.012	15 ± 2
			0.01 ± 0.004	0.029 ± 0.006	nd

Analysis of productivity for FHT (expressed as soluble protein) and CH3H (expressed as inclusion body) in dependence on the specific lactose uptake rate in HMS174(DE3) cultivations. Standard deviations were evaluated from triple measurements and calculated by error propagation

Indeed we found a correlating trend between the specific lactose uptake rate and productivity for both of the investigated model proteins. With lower specific lactose uptake rates, less target protein was produced, the effect being even more pronounced for inclusion bodies, where a reduction in the specific lactose uptake rate from 0.13 to 0.074 g/g/h led to a more than 10-fold decrease in specific product titers.

Nevertheless, it was unclear whether this reduction in expression resulted from a lower growth rate coming hand in hand with low specific sugar uptake rates or from less inducer available at lower specific lactose uptake rates. However, with the disaccharide both effects are linked. In order to decouple one from the other we again used IPTG (solely responsible for induction, but not growth) and glucose as non-inducing C-source. We varied the IPTG concentration at the same growth rate and also varied the growth rate at the same IPTG concentration. Once more, FHT was used as model protein—the results are shown in Table 3.

**Table 3:** FHT production in dependence on inducer (IPTG) concentration and growth rate

µ (h^−1^)	q_s,glu_ (g/g/h)	IPTG—conc. (mM)	Specific product titer (mg_P_/g_X_)
0.08 ± 0.014	0.29 ± 0.030	0.05	41 ± 1.3
0.07 ± 0.015	0.28 ± 0.046	0.5	38 ± 0.9
0.05 ± 0.004	0.16 ± 0.0013	0.5	35 ± 1.1
0.02 ± 0.006	0.066 ± 0.0009	0.5	27 ± 0.7

Analysis of productivity for FHT (expressed as soluble protein) in dependence on inducer (IPTG) concentration and growth rate (specific glucose uptake rate respectively) in HMS174(DE3) cultivations. FHT production does not decrease with less inducer but is lower with less glucose supplied. Standard deviations were evaluated from triple measurements and calculated by error propagation

Comparing production of target protein in HMS174(DE3) at the same growth rate, when either inducing with lactose (Table 2) or IPTG (Table 3), shows that induction with IPTG leads to higher product titers than induction with lactose, also if lactose is present in excess (38 mg product/g DCW and 27 mg product/g DCW respectively). We hypothesize that the reason for this is a different binding-dissociation constant of LacI to IPTG and lactose, causing different amounts of free repressor.

When comparing specific product titers in HMS174(DE3) and BL21(DE3) when induced with IPTG at the same growth rate of 0.07 h^−1^, the specific product titer is higher in HMS174(DE3) than in BL21(DE3) (38 mg product/g DCW and 25 mg product/g DCW, Tables 1 and 3). The reason for that could be an increased plasmid retention in HMS174(DE3). This has been investigated before by Marisch et al. who found that HMS174(DE3) had a higher plasmid retention than BL21(DE3). However, in contrast to our investigations they still observed higher productivity of target protein in strain BL21(DE3) [29].

Regarding the reason for decrease in protein expression at lower specific lactose uptake rates (less availability of inducer or lower growth rates) it can be stated that different amounts of IPTG (0.5 and 0.05 mM) did not cause a significant change in expression levels, in fact even slightly more protein was detected at the lower IPTG concentration. However, when the growth rate was turned down by supplying less glucose, FHT expression was clearly reduced, from 38 mg product/g DCW at a growth rate of 0.07 h^−1^ to 27 mg product/g DCW at a growth rate of 0.02 h^−1^. This strongly indicates that the reduction in productivity is a result of lower transcription and translation rates at lower growth rates but not a result of less inducer. Those results are in accordance with literature as the lacUV5 promoter (present in HMS174(DE3) and BL21(DE3)) has been found hardly titrable before [41–43].

## Conclusion

We showed that HMS174(DE3) represents an interesting and sustainable alternative to BL21(DE3) for the industrial production of enzymes. When only fed with lactose during induction, strain HMS174(DE3) clearly outcompetes BL21(DE3) in regard to biomass and product yields. However, more importantly, a lactose fed-batch with HMS174(DE3) even yields slightly higher specific product titers compared to a glucose fed-batch of BL21(DE3) at the same growth rate, induced with 0.5 mM IPTG. This strongly argues for employing strain HMS174(DE3) and growing it on inexpensive lactose during induction for the production of technical enzymes, especially at larger scales. In addition to cost reduction, our developed strategy further allows straight forward adjustment of product titers by regulation of lactose feeding rates.

## Methods

### Strains and plasmids

For cultivations *E. coli* strain BL21(DE3) (New England Biolabs, Ipswich, MA, USA) genotype: F^−^ *omp*T *hsd*S_B_ (r_B_^−^, m_B_^−^) *gal dcm* (DE3), strain JM109(DE3) (Promega, Madison, WI, USA) genotype: *end*A1 *rec*A1 *gyr*A96 *thi hsd*R17 (r_k_^–^ m_k_^+^) *rel*A1 *sup*E44 λ− ∆(*lac*-*pro*AB) [F′ *tra*D36 *pro*AB *lac*I^q^ZΔM15] (DE3) and strain HSM174(DE3) (kindly donated from Gerald Striedner, BOKU University of Natural Resources and Applied Life Sciences, Vienna, Austria) genotype: F^−^ *recA1 hsdR*(r_K12_^−^ m_K12_^+^) (DE3) (Rif^R^) were used. All three hosts carry the λ prophage in their genome allowing expression of target proteins from T7 promoters. Plasmids allowing such expression were employed: Either a pET21a plasmid (Novagen, Madison, WI, USA) encoding FHT from *M. domestica* or a pNIC plasmid (donation from Karolinska Institute, Stockholm, Sweden) encoding CH3H of *D. variabilis* were used. The gene encoding FHT was codon-optimized for expression in *E. coli* (Genscript, Piscataway, NJ, USA) and synthesized with the recognition sites for the endonucleases NdeI and XhoI by Invitrogen (Thermo Scientific, Waltham, MA, USA). The gene was cloned by restriction digest with NdeI and XhoI (New England Biolabs, Ipswich, MA, USA) into the pET21a(+) vector to form the corresponding FHTpET21a(+) construct. The plasmid for CH3H expression was a kind donation from Christina Divne and Rosaria Gandini (Karolinska Institute, Stockholm, Sweden). In brief: sub-cloning of the CH3H sequence into pNIC-CTHO was performed using the LIC-cloning methodology. The respective plasmids were transformed into strains HMS174(DE3), JM109(DE3) and BL21(DE3) by the heat shock method [44].

### Cultivations

#### Shake-flask cultivations

Shake flask cultivations were performed in a modified DeLisa minimal media [45] with a double amount of di-ammonium-hydrogen-phosphate for increased buffering and more nitrogen available. Additionally, the media was supplied with 0.1 g/L ampicillin. 1 L Erlenmeyer flasks filled with 100 mL modified DeLisa preculture media containing 8 g/L glucose was inoculated from frozen stocks (1.5 mL, − 80 °C) and grown over night at 37 °C, 230 rpm in an Infors HR Multitron shaker (Infors, Bottmingen, Switzerland). 50 mL of preculture were then added to 450 mL modified DeLisa media containing 9 g/L glucose and 9 g/L lactose. Flasks were incubated at 230 rpm and 30 °C for 23 h in the shaker. Samples were taken sterilely after 0, 3, 7 and 23 h and analyzed for OD_600_ which was determined with a Genesys 20 photometer (Thermo Scientific, Waltham, MA, USA). Samples were diluted with 0.9% NaCl solution to stay within the linear range of the photometer. From OD_600_ dry cell weight was calculated by an established correlation (DCW = 0.47 * OD_600_). For analysis of sugar contents 1 mL of the broth was transferred into a 1.5 mL plastic tube, centrifuged (4500*g*, 4 °C, 10 min) and frozen until HPLC measurement.

#### Cultivations in bioreactors

Cultivations were carried out in DAS-Gip parallel bioreactor systems (Eppendorf, Hamburg, Germany) with a volume of 2.7 L. The reactors were stirred at 1400 rpm and temperature was set to 35 °C or 30 °C respectively during induction. pH was monitored with pH-Sensor EasyFerm Plus (Hamilton, Reno, NV, USA) and kept constant at 7.2 by the addition of 12.5% NH_4_OH, the amount being monitored by DasGip MP8 Multi pump module (Eppendorf, Hamburg, Germany). The reactors were aerated at 2 vvm with a mixture of pressurized air and pure oxygen varying the ratio in a way that dO was kept above 30%. dO was measured with fluorescence dissolved oxygen electrode Visiferm DO425 (Hamilton, Reno, NV, USA). CO_2_ and O_2_ in the offgas were measured with a DAS-Gip GA gas analyzer (Eppendorf, Hamburg, Germany). Feed-rates were adjusted to control the specific sugar uptake rates. Therefore biomass was estimated using a soft-sensor tool as in [46].

All cultivations consisted of a Batch and Fed-Batch phase for biomass generation followed by an induced Fed-Batch for production. For the preculture a 2500 mL High-Yield Flask was filled with 500 mL of DeLisa minimal media containing 8 g/L glucose and 0.1 g/L ampicillin, inoculated from frozen stocks (1.5 mL, − 80 °C) and incubated for 16 h at 37 °C and 230 rpm in an Infors HR Multitron shaker (Infors, Bottmingen, Switzerland). For the Batch phase DeLisa Batch media with 20 g/L glucose was inoculated with a tenth of its volume of preculture. The end of the batch was detected by a drop in the CO_2_ signal and an increase in the dO signal and usually resulted in 8 g/L dry cell weight. Thereafter a fed-batch was started with a specific glucose uptake rate of 0.27 g/g/h until a dry cell weight concentration of 30 g/L was reached. Then induction was performed by either adding a pulse of IPTG to the desired concentration or a lactose pulse to 5 g/L. The glucose feedrate was kept at 0.27 g/g/h for 4 h allowing adaption to lactose. Thereafter, either the glucose feed was stopped and a lactose feed was started at the desired specific lactose uptake rate, or the glucose feedrate was turned down to the anticipated level. After further 6 h of induction the cultivations were stopped. Samples were taken after inoculation, at the end of the batch/beginning of the fed-batch, in the beginning of induction and every 2 h during induction. For analysis of cell dry weight 1 mL of cultivation broth was filled into pre-dried and pre-weighed 1.5 mL plastic tubes and centrifuged (4500*g*, 4 °C, 10 min). Supernatants were collected and frozen for HPLC sugar analysis. Pellets were washed with 1 mL of 0.9% NaCl solution before drying at 105 °C for 72 h. During induction additionally 20 mL fractions were centrifuged (4500*g*, 4 °C, 15 min) and the pellet was frozen at − 20 °C for subsequent product analysis.

### Analyses

#### Analysis of sugar in supernatant

Sugar concentrations were analyzed via HPLC (Thermo Scientific, Waltham, MA, USA) on a Supelcogel column (Supelco Inc., Bellefonte, Pennsylvania, USA) at a constant flow of 0.5 mL/min at 30 °C. The mobile phase consisted of 0.1% H_3_PO_4_ and sugars were detected with a Shodex RI-101 refractive index detector (DataApex, Prague, Czech Republic). Analysis of the chromatograms was performed using Chromeleon Software (Dionex, Sunnyvale, California, USA).

#### Analysis of soluble FHT protein

For analysis of soluble protein, the frozen cell pellets of 20 mL cultivation broth were resuspended in buffer A (20 mM Na-phosphate, 500 mM NaCl, 20 mM Imidazole, pH 7.4) to reach a dry cell weight concentration of 22 g/L. They were homogenized with PANDA homogenizer (GEA, Düsseldorf, Germany) for 10 passages at 1200 bar. For removal of cell debris a 40 mL aliquot was centrifuged (20.000*g*, 4 °C, 30 min). Product quantification was done with the preparative chromatography system ÄKTA pure (GE, Boston, Massachusetts, USA). The supernatant was loaded onto a 5 mL HisTrap FF column (GE Boston, Massachusetts, USA) which was equilibrated with buffer A. After loading the column was washed until the UV signal was constant. Target protein was eluted in a step gradient to 100% buffer B (20 mM Na–phosphate, 500 mM NaCl, 500 mM imidazole, pH 7.4). Eluate was collected and protein content was determined by a Bradford assay. Bradford reagent was purchased from Sigma Aldrich (Sigma-Aldrich, Vienna, Austria) and bovine serum albumin (Sigma-Aldrich, Vienna, Austria) was used as standard. Samples were diluted with buffer A to stay in the linear range (0.1–0.8 absorption units) of the Genesys 20 photometer (Thermo Fisher Scientific, Waltham, MA, USA). Purity was checked by SDS-PAGE analysis. Samples were mixed 1:1 with a 2× concentrated Laemmli buffer [47] and kept at 95 °C for 10 min. After centrifugation (14,000 rpm, 21 °C, 5 min) the samples were loaded on an Any kD™ Mini-PROTEAN^®^ TGX™ Precast Protein Gel, 10-well, 30 µl (Bio-Rad, Vienna, Austria). 5 μL of PageRuler™ Plus Prestained Protein Ladder (Thermo Fisher Scientific, Waltham, MA, USA) were used as a standard. Gels were run in SDS buffer (3.03 g/L Tris, 7.2 g/L glycine, 1.0 g/L SDS) in a Mini-PROTEAN^®^ Tetra Vertical Electrophoresis Cell (Bio-Rad, Hercules, CA, USA) at a constant voltage of 180 V for 35 min. Gels were stained with Coomassie Sensitive stain (50 g/L aluminium sulfate (14–18 hydrate), 100 mL/L ethanol, 23.5 mL/L orthophosphoric acid, 0.2 g/L Coomassie blue G250) over night, washed with water and analyzed with Gel Doc XR system and ImageLab software (Bio-Rad, Hercules, CA, USA).

#### Analysis of inclusion bodies (CH3H)

Analysis of inclusion bodies was performed by reverse phase HPLC techniques as in [19].

## Additional file

**Additional file 1: Figure S1.** FHT production by HMS174(DE3) during the characterization-cultivation and comparison to IPTG induced BL21(DE3) cells. SDS-PAGE showing: Protein ladder (A), homogenized HMS174(DE3) cells before induction (B), homogenized HMS174(DE3) cells after 10 h induction by lactose (C), BL21(DE3) cells before induction (D), homogenized BL21(DE3) cells after 10 h induction by IPTG (E), FHT-standard (F).

## Abbreviations

IPTG: isopropyl-β-d-thiogalactopyranosid
q_s,glu_: specific glucose uptake rate
q_s,lac_: specific lactose uptake rate
FHT: flavanone 3-hydroxylase
CH3H: chalcone-3-hydroxylase
DCW: dry cell weight
OD_600_: optical density at 600 nm
vvm: volume of gas per volume of medium per minute
dO: dissolved oxygen
