# Supplementary figures and images for: E. coli HMS174(DE3) is a sustainable alternative to BL21(DE3)
Source: Microb Cell Fact. 2018 Oct 30;17:169. doi: 10.1186/s12934-018-1016-6 (PMC6206895; doi:10.1186/s12934-018-1016-6)

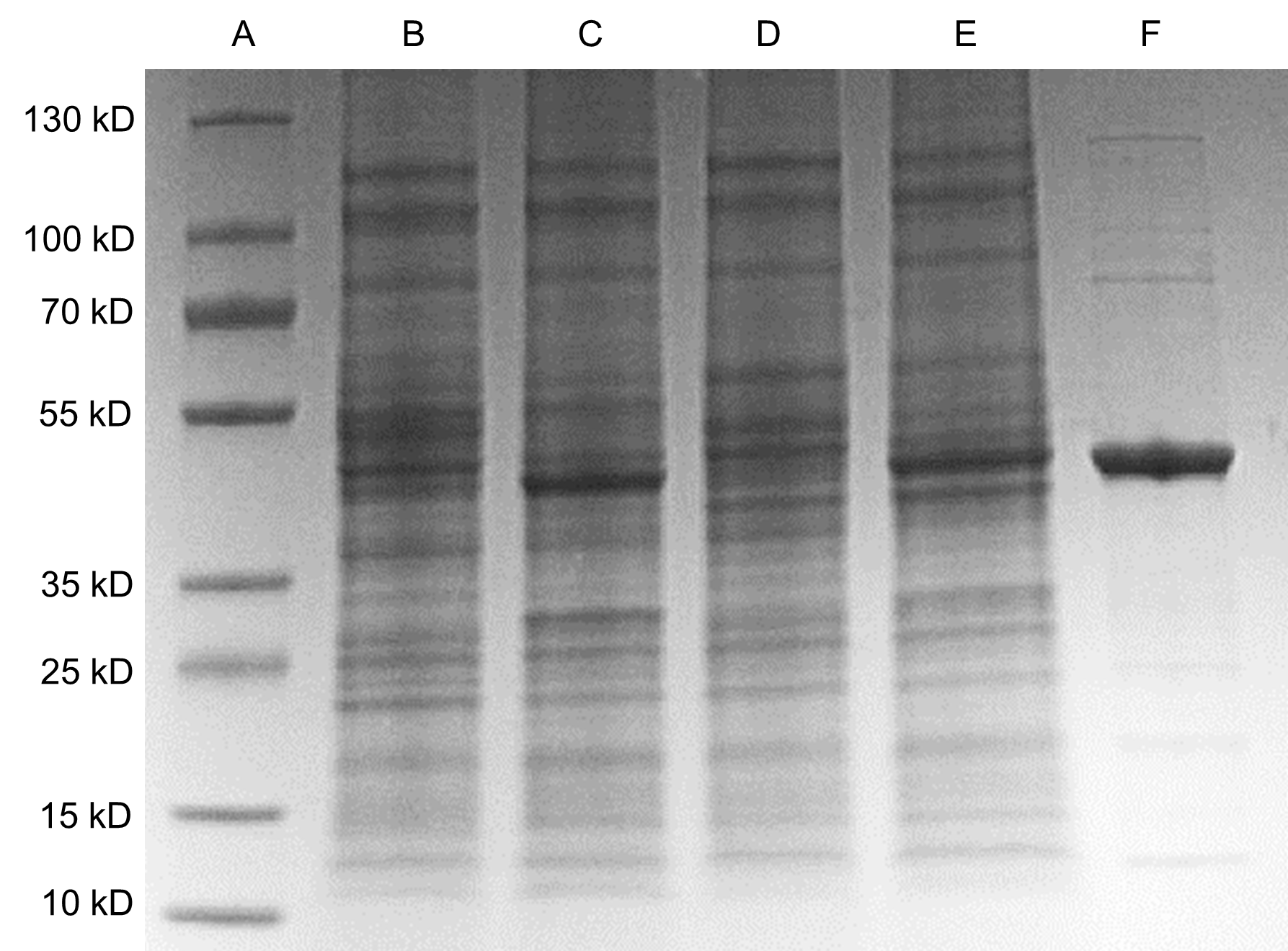

Supplement: Supplementary file 1 — Additional file 1: Figure S1. FHT production by HMS174(DE3) during the characterization-cultivation and comparison to IPTG induced BL21(DE3) cells. SDS-PAGE showing: Protein ladder (A), homogenized HMS174(DE3) cells before induction (B), homogenized HMS174(DE3) cells after 10 h induction by lactose (C), BL21(DE3) cells before induction (D), homogenized BL21(DE3) cells after 10 h induction by IPTG (E), FHT-standard (F). [file 12934_2018_1016_MOESM1_ESM.tif]
